# Sex disparities in dietary intake across the lifespan: the case of Lebanon

**DOI:** 10.1186/s12937-020-00543-x

**Published:** 2020-03-26

**Authors:** Lara Nasreddine, Marie Claire Chamieh, Jennifer Ayoub, Nahla Hwalla, Abla-Mehio Sibai, Farah Naja

**Affiliations:** 1 Department of Nutrition and Food Science, Faculty of Agricultural and Food Sciences, American University of Beirut, Beirut, Lebanon; 2 Department of Epidemiology and Population Health, Faculty of Health Sciences, American University of Beirut, Beirut, Lebanon

**Keywords:** Sex-based differences, Dietary intakes, Food consumption patterns, Food groups, Macronutrients, Micronutrients, Nutrition, Lebanon

## Abstract

**Background:**

Little is known about sex-based dietary differences in middle-income countries, particularly those undergoing the nutrition transition. This study aims at examining sex disparities in energy and macronutrients’ intakes, food consumption patterns, and micronutrients’ adequacy in Lebanon, while adopting a life course approach.

**Methods:**

Data were derived from a national cross-sectional survey conducted in Lebanon in 2008/2009. The study sample consisted of 3636 subjects: 956 children and adolescents aged 6–19.9 years; 2239 adults aged 20–59.9 years and 441 older adults aged above 60 years. At the households, trained nutritionists conducted face-to-face interviews with participants to complete a sociodemographic questionnaire and one 24-h diet recall. Food items were categorized into 25 food groups. The Nutritionist Pro software was used for the analysis of dietary intake data and the estimation of energy, macronutrients’, and micronutrients’ intakes.

**Results:**

In all age groups, males had significantly higher energy intakes, while females had significantly higher fiber intakes. In addition, in adolescents aged 12–19.9 years, females had higher fat intakes as compared to males (37.02 ± 0.6% vs 35.03 ± 0.61%), and in adults aged 20–59.9 years, females had significantly higher total fat (37.73 ± 0.33% vs 36.45 ± 0.38%) and saturated fat intakes (11.24 ± 0.15% vs 10.45 ± 0.18%). These differences in macronutrient intakes were not observed in younger children nor in older adults. Sex-based differences in food groups’ intakes were also observed: men and boys had significantly higher intakes of red and processed meat, bread, fast food, soft drinks, and alcohol, while girls and women had higher intakes of fruits, vegetables, milk, and sweets. In all age groups, females had lower micronutrient intakes compared to males, including calcium, iron, and zinc.

**Conclusions:**

This study identified sex-specific priorities that ought to be tackled by context-specific interventions to promote healthier diets in Lebanon. The fact that sex-based differences in nutrient intakes and food consumption patterns were the most noticeable in the adolescent and adult years, hence women’s reproductive years call for concerted efforts to improve nutrition for women and girls as this would lay the foundation not only for their future education, productivity, and economic empowerment, but also for the health of future generations.

## Introduction

Goal 5 of the United Nations’ Sustainable Development Goals (SDGs) aims to “achieve gender equality and empower all women and girls” around the world. While women and girls often have increased nutritional needs during the lifecycle, social norms in many parts of the world frequently lead to gender inequalities in nutrition, which tend to disfavor females [1]. Worldwide, women and girls continue to be twice as likely to suffer from all forms of malnutrition compared to their male counterparts, highlighting the need for a better understanding of sex disparities in food consumption and dietary intakes as a determinant of health [1]. In this manuscript, and based on the recommendations of the Institute of Medicine, the term “sex” is used as a biological classification determined by sex chromosomes, while the term gender is used when referring to behavioral, cultural, or psychological traits typically associated with one sex [2, 3].

Evidence stemming from different parts of the world depicts conflicting pictures of sex-based discrepancies in diet and nutrition [4–7]. Studies conducted in developing, low income countries, showed that women and girls have lower intakes of nutrient-dense foods such as meats, eggs, milk, pulses, fruits, and vegetables and a higher risk for micronutrients and chronic energy deficiencies compared to males [8]. However, in Western developed countries, studies have shown that females tend to make healthier food choices and consume higher amounts of fruits, vegetables, and dietary fiber compared to males [9]. Little is known about sex-based dietary differences in middle-income countries, particularly those undergoing the nutrition transition, with its characteristic shifts in diet and lifestyle [5]. This may be particularly true for the Eastern Mediterranean Region (EMR), which has witnessed rapid changes in food consumption habits and dietary practices over the past decades. The region is also characterized by a double burden of malnutrition with strong female vs. male disparities: Women and girls in the EMR have a higher burden of micronutrient deficiencies while also being approximately three times more prone to obesity compared to males [10–12]. Apart from sex-based physiological and biological differences, a variety of external factors may explain these gender disparities, including the non-egalitarian household and social roles in the region [13–16].

Lebanon, a small country of the EMR, has made substantial achievements with regard to gender equality and empowerment [17, 18] over the past 10 years. However, similar to other countries of the region, the country still harbors a set of traditions and social norms that may result in unequal gender roles both within the household and in the society [5]. These include unequal division of household chores, lower expectations for women regarding education and/or professional achievements, and gender-based constraints on physically active leisure activities in addition to social legislations disfavoring women [5, 19]. It is important to understand whether these non-egalitarian social roles in Lebanon are also associated with disparities in diet and food consumption, particularly that sex-based disparities in the burden of micronutrient deficiencies’ and obesity prevalence were previously reported [12, 20]. Interestingly, previous studies in Lebanon showed that sex differentials in obesity prevalence were not static, but were rather dynamic across the lifespan. For instance, while obesity rates did not differ between boys and girls in young children, significant sex-based disparities were noted in the adolescent age group, with the prevalence of obesity being significantly higher in boys compared to girls [21]. Among adults, men showed higher prevalence rates at the younger age groups (20–49 years), while women showed higher prevalence rates in older age groups (50 years and above). These differentials were most apparent in obesity class II and class III with women being significantly more likely than men to show this type of excessive adiposity [20].

Acknowledging the need for a better understanding of gender-based differences in dietary intakes, this study aims at examining sex-based disparities in energy and macronutrients’ intakes, food consumption patterns, and micronutrients’ adequacy in Lebanon, while adopting a life course approach. The characterization of sex-based disparities in dietary intakes would be useful to set priorities and inform the development and implementation of comprehensive interventions aimed at reducing inequalities in food and nutrient intakes across the lifespan and subsequently enhancing the population’s nutritional status and health profile [4].

## Methods

The data for the present study were derived from the national cross-sectional food consumption survey conducted in Lebanon between May 2008 and August 2009. Details about the design and protocol of this survey are found elsewhere [22, 23]. In brief, the survey was based on the sampling frame provided by the Ministry of Social Affairs/Central Administration of Statistics in collaboration with United Nations Development Programme (UNDP). Efforts were exerted to recruit a nationally representative sample with age, sex, and district distributions proportionate to that of the Lebanese population [24, 25]. The study sample consisted of randomly selected households, based on stratified cluster sampling: the strata were the Lebanese governorates, the clusters were selected further at the level of districts, urban, and rural areas, and the housing units constituted the primary sampling units. One adult from each household and one child/adolescent from every other household were selected from the household roster, excluding pregnant and lactating women and subjects with mental disabilities. The study sample consisted of 3636 subjects (956 children and adolescents aged 6–19.9 years; 2239 adults aged 20–59.9 years and 441 older adults aged above 60 years) (response rate: 89.3%). The design and conduct of the survey were approved by the Institutional Review Board of the American University of Beirut, and informed consents from adults/parents and informed assents from children and adolescents were obtained prior to enrollment in the study.

At the households, trained nutritionists conducted face-to-face interviews with participants to complete a sociodemographic questionnaire and one 24-h diet recall (24-HR). The questionnaire covered information related to sex (male, female), age (in years), and governorate of current residence (Beirut, Mount Lebanon, North, South, Bekaa, and Nabatieh). For children and adolescents aged between 6 and 19.9 years, questions about mother’s and father’s educational levels (primary education or less, up to high school education, and university degree or higher) were included in the questionnaire. For adults aged 20 years and above, sociodemographic information collected were related to crowding index, marital status (single, married, or divorced/separated/widowed), educational level (primary education or less, up to high school education, and university degree or higher), and employment status (employed, not employed, housewife, or student). In this survey, crowding index was used as a proxy for socioeconomic status. This index is calculated as the ratio of the number of people living in the household over the number of rooms, excluding the kitchen and bathrooms. Several epidemiological studies have associated a high household crowding index with low socioeconomic status [26, 27].

Dietary intake was examined using the 24-HRs referring to dietary intake consumption during the previous 24 h. The 24-HRs in this study were carried out using the Multiple Pass Food Recall 5-step approach, developed by the United States Department of Agriculture (USDA) [28]. This approach has consistently showed attenuation in the 24-HRs’ limitations [29]. The five steps followed included 1) quick food list recall, 2) forgotten food list probe, 3) time and occasion at which foods were consumed, 4) detailed overall cycle, and 5) final probe review of the foods consumed. For children up to 12 years of age, the mother was considered as proxy. In case the responsibility for feeding of the child was shared with another caregiver (father, helper, or other), the mother was encouraged to consult with the caregiver to obtain additional/complementary details about the dietary intake of the child. For adolescents (aged between 12 and 19.9 years), participants were interviewed directly to complete the 24-HRs and caregivers were consulted if further details were needed regarding ingredients, methods of preparation, and portion sizes. The latter was determined using quantification tools [30] and which included standard measuring cups and spoons, household measures, as well as food photos and models of single servings of commonly consumed food items. The nutritionists who were collecting the dietary intake data were specifically trained to maintain a neutral attitude and avoid leading questions.

The Nutritionist Pro software (version 5.1.0, 2014, First Data Bank, Nutritionist Pro, Axxya Systems, San Bruno, CA) was used for the analysis of dietary intake data and the estimation of energy, macronutrients’, and micronutrients’ intakes. For composite and mixed dishes, standardized recipes were added to the Nutritionist Pro Software using single food items. Within the Nutritionist Pro, the USDA database was selected for analysis (SR 24, published September 2011). Food composition of specific Lebanese foods (not included in the Nutritionist Pro software database) was obtained from local food composition tables [31]. In addition to energy, macronutrients, and micronutrients, the dietary intake data was also used to obtain information about food groups intakes. Food items were categorized into the following 25 food groups: bread, cereals, legumes, starchy vegetables, vegetables, chips and salty crackers, nuts and seeds, milk, milk derivatives, sweetened milk, red meat, processed meat, poultry, fish, eggs, fruits, fresh fruit juices, sweets, added sugars, sugar sweetened beverages, hot beverages (coffee, tea), alcoholic beverages, added fats and oils, fast food, and miscellaneous. Food items included within each of the aforementioned food groups are outlined in Appendix A.

### Statistical analyses

The data for this study were presented and compared between sexes among the various age groups (6–19.9 years, 20–59.9 years, and ≥ 60 years). In addition, data for adolescents (aged 12–19.9 years) were examined and presented separately in Appendix B. Sociodemographic and dietary intake variables were described by means ± standard error (SE) and proportions and were compared between males and females using t-test and chi square test, for continuous and categorical variables, respectively. Macronutrients’ and food groups’ intakes were expressed as percent of total energy intake (% EI). Micronutrients’ intakes were presented as means ± SE, as well as percent of the population meeting 2/3rd of the Recommended Daily Allowance (RDA). The means ± SE of dietary intakes were adjusted for differences in sociodemographic variables among males and females, using Analysis of Covariance (ANCOVA). In the latter, the sociodemographic covariates included were those that showed statistical difference between males and females. The Statistical Package for Social Sciences 22.0 (SPSS for Windows, 2013, Chicago: SPSS Inc.) was used for the statistical analyses. All statistical analyses were two-tailed and a *p*-value < 0.05 was considered statistically significant.

## Results

Participants with missing dietary intake data were not included in the study (*n* = 149). In addition, a total of 93 subjects were excluded as outliers, based on total energy intake and using the interquartile range method [32]. Hence the number of participants included in this study was *n* = 3394. Sociodemographic characteristics of the study population by age and sex are presented in Table 1. Among children and adolescents, the proportions of participants aged 6–11.9 and 12–19.9 years were 42.8 and 57.2% respectively. As for governorate, the highest proportions of participants came from Mount Lebanon (37.1%) and the North (23.5%), with the lowest proportions being from Beirut (6.7%) and Nabatieh (5.4%). Most of the children and adolescents in this age group belonged to households with a crowding index ≥1 (80.9%). The distributions of the mother and father’s education levels were similar, whereby the majority had ‘up to a high school’ level (father’s education: 53.4%, mother’s education: 59.3%) (Table 1). A comparison of sociodemographic characteristics between males and females in this age group showed a significant difference in the distribution across governorates (*p* = 0.003) (Table 1). Among adults aged 20 years and older, the study population consisted of 83.2% adults aged between 20 and 59.9 years and 16.8% older than 60 years. Over 40% of subjects were from Mount Lebanon (43.7%) and 62.2% had a crowding index ≥1. Sixty percent of participants (60.5%) were married, 31.2% single, and 8.3% divorced, separated, or widowed. As for the education level, almost one in two participants (49.7%) had ‘up to a high school’ level and 29.1% had a university degree or higher. Similarly, one in two participants (49.6%) reported being ‘employed’, while 10.4% were ‘not employed’, 33% were housewives, and 7% were students (Table 1). The comparison between males and females among adults in the study population showed significant differences related to all variables, except for education where the distributions of males and females across the three levels of education were not statistically different. More specifically, the proportion of older adults (≥60 years) was higher among males than females (18.8% vs 15.1%, *p* = 0.011). As for governorates, Beirut and Mount Lebanon had greater proportions of males, while Bekaa and Nabatieh had greater proportions of females. The proportion of females with a crowding index ≥1 was higher than males (64.4% vs 59.6%, *p* = 0.013). Furthermore, a higher proportion of females was divorced, separated, or widowed as compared to males (12.2% vs 3.5%, p <0.001). As for employment status, 75.4% of males vs 28.5% of females reported being ‘employed’ (p <0.001) (Table 1).

**Table 1:** Sociodemographic characteristics by gender of the total study population (*n* = 3394)

Children and Adolescents				
	Total (*n* = 865)	Males (*n* = 437)	Females (*n* = 428)	*p*-value^*^
Age (years)				
6–11.9	370 (42.8)	193 (44.2)	177 (41.4)	0.404
12–19.9	495 (57.2)	244 (55.8)	251 (58.6)	
Governorate				
Beirut	56 (6.7)	34 (8.1)	22 (5.2)	**0.003**
Mount Lebanon	311 (37.1)	178 (42.6)	133 (31.6)	
North	197 (23.5)	92 (22.0)	105 (24.9)	
South	117 (13.9)	47 (11.2)	70 (16.6)	
Bekaa	113 (13.5)	48 (11.5)	65 (15.4)	
Nabatieh	45 (5.4)	19 (4.5)	26 (6.2)	
Crowding index				
< 1 Person/Room	165 (19.1)	89 (20.4)	76 (17.8)	0.337
≥ 1 Person/Room	699 (80.9)	348 (79.6)	351 (82.2)	
Father’s education level				
Primary or lower	236 (30.8)	115 (29.9)	121 (31.7)	0.467
Up to high school	409 (53.4)	213 (55.5)	196 (51.3)	
University or higher	121 (15.8)	56 (14.6)	65 (17.0)	
Mother’s education level				
Primary or lower	192 (24.3)	101 (25.8)	91 (22.9)	0.405
Up to high school	468 (59.3)	233 (59.4)	235 (59.2)	
University or higher	129 (16.3)	58 (14.8)	71 (17.9)	
Adults				
	Total (*n* = 2529)	Males (*n* = 1141)	Females (*n* = 1388)	*p*-value*
Age (years)				
20–59.9	2105 (83.2)	926 (81.2)	1179 (84.9)	**0.011**
≥ 60	424 (16.8)	215 (18.8)	209 (15.1)	
Governorate				
Beirut	281 (11.1)	153 (13.4)	128 (9.2)	**< 0.001**
Mount Lebanon	1104 (43.7)	523 (45.8)	581 (41.9)	
North	420 (16.6)	188 (16.5)	232 (16.7)	
South	311 (12.3)	130 (11.4)	181 (13.0)	
Bekaa	296 (11.7)	106 (9.3)	190 (13.7)	
Nabatieh	117 (4.6)	41 (3.6)	76 (5.5)	
Crowding index				
< 1 Person/Room	951 (37.8)	459 (40.4)	492 (35.6)	**0.013**
≥ 1 Person/Room	1567 (62.2)	677 (59.6)	890 (64.4)	
Marital status				
Single	788 (31.2)	429 (37.6)	359 (25.9)	**< 0.001**
Married	1530 (60.5)	671 (58.9)	859 (61.9)	
Divorced, separated, or widowed	209 (8.3)	40 (3.5)	169 (12.2)	
Education level				
Primary or lower	534 (21.1)	253 (22.2)	281 (20.2)	0.488
Up to high school	1258 (49.7)	562 (49.3)	696 (50.1)	
University or higher	737 (29.1)	326 (28.6)	411 (29.6)	
Employment status				
Employed	1254 (49.6)	859 (75.4)	395 (28.5)	**< 0.001**
Not employed^**^	262 (10.4)	203 (17.8)	59 (4.3)	
Housewife	835 (33.0)	1 (0.1)	834 (60.1)	
Student	177 (7.0)	77 (6.8)	100 (7.2)	

Values in this table represent n (%)

^*^p-value derived from chi square test

^**^Not employed included not working, does not want to work, looking for work, or retired

**Bolded** numbers are significant at *p* < 0.05

Energy and macronutrients’ intakes (expressed as percentage of total energy) of the study population are presented in Table 2. Among children and adolescents, energy intake was 1999.81 ± 27.17 kcal and the percentage contributions of macronutrients to energy were as follows: Carbohydrates: 51.57 ± 0.34%; proteins: 13.24 ± 0.13%; fat: 36.11 ± 0.31%. Saturated fat and total sugars made up 10.86 ± 0.17% and 12.01 ± 0.27% respectively. Fiber intake was estimated at 7.47 ± 0.13 g/1000 kcal. In this age group, comparison between males and females showed that males had a higher energy intake, while females had higher fiber intakes (Table 2). When adolescents (12–19.9 years) were examined separately, fat intake was shown to be significantly different among sexes, in addition to energy and fiber intakes. More specifically, females had higher fat intakes as compared to males (37.02 ± 0.6% vs 35.03 ± 0.61%) (Appendix B-Table 1). Among adults between 20 and 59.9 years, the contributions of macronutrients to energy were as follows: 48.8 ± 0.25% for carbohydrates, 14.66 ± 0.11% for proteins, and 37.09 ± 0.23% for fat. In this age group, while males had significantly higher energy intakes, females had higher fat, saturated fat, and fiber intakes. Among older adults, the contributions of macronutrients to total energy intake were 49.48 ± 0.58% for carbohydrates, 14.88 ± 0.25% for proteins, and 36.27 ± 0.52% for fat. In this age group, the comparison of energy and macronutrients’ intakes between males and females showed similar results as those for children and adolescents, whereby males had higher energy intakes while females had higher fiber intakes (Table 2).

**Table 2:** Energy and macronutrient intake by gender in the study population (n = 3394)

	Total (*n* = 3394)	Males (*n* = 1578)	Females (*n* = 1816)	p-value^*^
		**Mean ± SE**		
**Children and adolescents (6–19.9 years)**				
Energy (kcal)	1999.81 ± 27.17	2265.55 ± 38.71	1734.06 ± 38.48	**< 0.001**
Carbohydrate (%EI)	51.57 ± 0.34	51.59 ± 0.47	51.53 ± 0.47	0.923
Protein (%EI)	13.24 ± 0.13	13.31 ± 0.17	13.16 ± 0.17	0.539
Fat (%EI)	36.11 ± 0.31	35.78 ± 0.44	36.44 ± 0.44	0.300
Saturated fat (%EI)	10.86 ± 0.17	10.76 ± 0.23	10.95 ± 0.23	0.565
Oleic Acid (%EI)	9.64 ± 0.2	9.68 ± 0.27	9.59 ± 0.27	0.815
Linolenic Acid (%EI)	0.13 ± 0	13 ± 0	0.13 ± 0	0.552
Linoleic Acid (%EI)	4.73 ± 0.11	4.79 ± 0.16	4.65 ± 0.15	0.547
Total Sugar (%EI)	12.01 ± 0.27	11.63 ± 0.38	12.38 ± 0.38	0.173
Dietary fibers (g/1000 kcal)	7.47 ± 0.13	6.93 ± 0.19	8.01 ± 7.65	**< 0.001**
**Adults (20–59.9 years)**				
Energy (kcal)	1983.17 ± 18.26	2309.95 ± 29.98	1656.39 ± 26.07	**< 0.001**
Carbohydrate (%EI)	48.8 ± 0.25	48.82 ± 0.42	48.77 ± 0.36	0.940
Protein (%EI)	14.66 ± 0.11	14.68 ± 0.19	14.63 ± 0.16	0.868
Fat (%EI)	37.09 ± 0.23	36.45 ± 0.38	37.73 ± 0.33	**0.017**
Saturated fat (%EI)	10.84 ± 0.11	10.45 ± 0.18	11.24 ± 0.15	**0.002**
Oleic Acid (%EI)	11.85 ± 0.15	11.75 ± 0.25	11.95 ± 0.22	0.587
Linolenic Acid (%EI)	0.15 ± 0	0.15 ± 0.01	0.16 ± 0.01	0.252
Linoleic Acid (%EI)	4.96 ± 0.09	4.87 ± 0.14	5.05 ± 0.12	0.368
Total Sugar (%EI)	10.85 ± 0.18	10.69 ± 0.3	11.01 ± 0.26	0.455
Dietary fibers (g/1000 kcal)	8.82 ± 0.11	8.32 ± 0.18	9.31 ± 0.16	**< 0.001**
**Older adults (≥60 years)**				
Energy (kcal)	1628.19 ± 34.1	1861.8 ± 62.71	1394.59 ± 63.77	**< 0.001**
Carbohydrate (%EI)	49.48 ± 0.58	48.28 ± 1.07	50.68 ± 1.09	0.187
Protein (%EI)	14.88 ± 0.25	14.54 ± 0.45	15.22 ± 0.46	0.380
Fat (%EI)	36.27 ± 0.52	37.35 ± 0.96	35.2 ± 0.98	0.189
Saturated fat (%EI)	10.5 ± 0.23	10.42 ± 0.42	40.58 ± 0.43	0.826
Oleic Acid (%EI)	12.5 ± 0.34	12.78 ± 0.63	12.22 ± 0.64	0.603
Linolenic Acid (%EI)	0.17 ± 0.01	0.15 ± 0.01	0.18 ± 0.01	0.167
Linoleic Acid (%EI)	4.35 ± 0.18	4.73 ± 0.33	3.98 ± 0.33	0.178
Total Sugar (%EI)	10.72 ± 0.4	11.0 ± 0.73	10.45 ± 0.75	0.654
Dietary fibers (g/1000 kcal)	10.63 ± 0.26	9.6 ± 0.47	11.65 ± 0.48	**0.011**

**Abbreviations:***EI* energy intake; *g* grams; *kcal* calories; *SE* standard error

Values in this table represent mean ± SE

Means were adjusted for variables found to be significantly associated with gender, as per Table 1

^*^p values were obtained from comparison of adjusted means using ANCOVA

**Bolded** numbers are significant at *p* < 0.05

The contributions of the various food groups to energy intakes by sex among different age groups are presented in Table 3. Among children and adolescents, the percent contributions of the following food groups were higher in males as compared to females: red meat (5.53 ± 0.47% vs 3.96 ± 0.47%), sugar sweetened beverages (7.16 ± 0.34% vs 5.73 ± 0.34%), and fast food (3.37 ± 0.42% vs 1.65 ± 0.42%). On the other hand, females had higher contributions of whole fruits to their total energy intake than males (4.33 ± 0.28% vs 2.71 ± 0.28%). Among adolescents (12–19.9 years), the contributions of bread, processed meat, and fast food to total energy intake were higher among males than females, while those for nuts and seeds, whole fruits, and sweets were higher among females (Appendix B-Table 2). Among adults (20–59.9 years), further differences in food groups’ contributions to total energy intakes were observed, whereby males had greater intakes of bread, red meat, sugar sweetened beverages, alcoholic beverages, and fast food, while females had higher intakes of vegetables, milk and milk derivatives, whole fruits, sweets, and hot beverages. Among older adults (≥60 years), fewer differences in food groups’ intakes were observed as compared to younger age groups, with only vegetables intakes being higher among females (Table 3).

**Table 3:** Percent contribution of various food groups to total every intake by gender (n = 3394)

	Total (*n* = 3394)	Males (*n* = 1578)	Females (*n* = 1816)	*p*-value^*^
		Mean %EI ± SE		
Children and adolescents (6–19.9 years)				
Bread	16.67 ± 0.44	17.35 ± 0.62	15.98 ± 0.62	0.122
Cereals	14.77 ± 0.63	15.56 ± 0.89	13.99 ± 0.89	0.216
Legumes	2.69 ± 0.30	2.48 ± 0.43	2.9 ± 0.43	0.484
Starchy Vegetables	5.14 ± 0.29	4.85 ± 0.41	5.44 ± 0.41	0.311
Vegetables	4.94 ± 0.30	4.37 ± 0.43	5.52 ± 0.43	0.060
Chips & Salty Crackers	4.12 ± 0.27	4.03 ± 0.39	4.21 ± 0.39	0.750
Nuts & Seeds	1.67 ± 0.24	1.22 ± 0.34	2.11 ± 0.34	0.066
Dairy Products				
Milk	1.87 ± 0.16	1.63 ± 0.22	2.12 ± 0.22	0.123
Milk Derivatives	6.18 ± 0.29	5.88 ± 0.41	6.48 ± 0.41	0.304
Milk Sweetened	0.31 ± 0.08	0.32 ± 0.12	0.31 ± 0.12	0.956
Meat, Processed Meat, Poultry, Fish, Eggs				
Red Meat	4.74 ± 0.33	5.53 ± 0.47	3.96 ± 0.47	**0.020**
Processed Meat	2.0 ± 0.20	2.34 ± 0.29	1.64 ± 0.29	0.091
Poultry	3.60 ± 0.31	3.53 ± 0.44	3.66 ± 0.44	0.835
Fish	0.68 ± 0.16	0.77 ± 0.22	0.60 ± 0.22	0.573
Eggs	1.07 ± 0.14	1.14 ± 0.20	0.98 ± 0.20	0.578
Fruits, Total				
Whole Fruits	3.52 ± 0.20	2.71 ± 0.28	4.33 ± 0.28	**< 0.001**
Fresh Juices (100% fruit juices)	0.10 ± 0.03	0.08 ± 0.04	0.12 ± 0.04	0.514
Sweets & Added Sugars				
Sweets	9.20 ± 0.43	8.46 ± 0.61	9.94 ± 0.61	0.092
Added Sugars, Jams, Honey, Molasses	1.82 ± 0.11	1.77 ± 0.16	1.88 ± 0.16	0.617
Sugar Sweetened Beverages	6.44 ± 0.24	7.16 ± 0.34	5.73 ± 0.34	**0.003**
Hot Beverages (Coffee, Tea)	0.11 ± 0.01	0.11 ± 0.02	0.1 ± 0.02	0.888
Alcoholic Beverages	0.15 ± 0.06	0.16 ± 0.08	0.13 ± 0.08	0.817
Added Fats & Oils	4.69 ± 0.24	4.23 ± 0.35	5.16 ± 0.35	0.058
Fast Food	2.51 ± 0.29	3.37 ± 0.42	1.65 ± 0.42	**0.004**
Miscellaneous	1.01 ± 0.09	0.96 ± 0.13	1.07 ± 0.13	0.549
Adults (20–59.9 years)				
Bread	17.21 ± 0.34	19.28 ± 0.54	15.14 ± 0.49	**< 0.001**
Cereals	15.94 ± 0.50	16.39 ± 0.79	15.49 ± 0.72	0.429
Legumes	3.51 ± 0.24	4.07 ± 0.39	2.95 ± 0.35	0.043
Starchy Vegetables	3.90 ± 0.17	3.72 ± 0.27	4.09 ± 0.25	0.348
Vegetables	8.32 ± 0.28	6.10 ± 0.45	10.55 ± 0.41	**< 0.001**
Chips & Salty Crackers	0.93 ± 0.09	0.74 ± 0.14	1.12 ± 0.13	0.059
Nuts & Seeds	2.46 ± 0.20	2.40 ± 0.31	2.52 ± 0.28	0.795
Dairy Products				
Milk	0.99 ± 0.08	0.70 ± 0.13	1.29 ± 0.11	**0.001**
Milk Derivatives	7.05 ± 0.22	6.50 ± 0.36	7.59 ± 0.32	**0.033**
Milk Sweetened	0.26 ± 0.06	0.20 ± 0.09	0.31 ± 0.08	0.356
Meat, Processed Meat, Poultry, Fish, Eggs				
Red Meat	6.46 ± 0.29	7.03 ± 0.46	5.89 ± 0.42	**0.086**
Processed Meat	0.79 ± 0.08	0.87 ± 0.12	0.72 ± 0.11	0.406
Poultry	4.21 ± 0.24	4.55 ± 0.38	3.87 ± 0.35	0.224
Fish	1.22 ± 0.13	1.49 ± 0.20	0.95 ± 0.18	0.061
Eggs	0.74 ± 0.07	0.86 ± 0.10	0.61 ± 0.10	0.095
Fruits, Total				
Whole Fruits	4.03 ± 0.15	3.30 ± 0.23	4.76 ± 0.21	**< 0.001**
Fresh Juices (100% fruit juices)	0.22 ± 0.03	0.24 ± 0.05	0.20 ± 0.05	0.528
Sweets & Added Sugars				
Sweets	6.70 ± 0.27	4.81 ± 0.44	8.60 ± 0.40	**< 0.001**
Added Sugars, Jams, Honey, Molasses	1.65 ± 0.07	1.56 ± 0.11	1.75 ± 0.10	0.242
Sugar Sweetened Beverages	4.0 ± 0.15	4.42 ± 0.24	3.57 ± 0.22	**0.012**
Hot Beverages (Coffee, Tea)	0.65 ± 0.05	0.46 ± 0.08	0.83 ± 0.07	**0.001**
Alcoholic Beverages	0.74 ± 0.08	1.30 ± 0.14	0.19 ± 0.12	**< 0.001**
Added Fats & Oils	4.32 ± 0.16	4.62 ± 0.26	4.01 ± 0.24	0.110
Fast Food	2.56 ± 0.23	3.20 ± 0.37	1.93 ± 0.33	**0.016**
Miscellaneous	1.15 ± 0.10	1.20 ± 0.17	1.10 ± 0.15	0.675
Older adults (≥ 60 years)				
Bread	20.82 ± 0.83	21.88 ± 1.48	19.77 ± 1.63	0.424
Cereals	14.67 ± 1.07	12.26 ± 1.90	17.07 ± 2.09	0.156
Legumes	3.59 ± 0.53	4.93 ± 0.95	2.25 ± 1.04	0.112
Starchy Vegetables	2.68 ± 0.34	2.89 ± 0.61	2.46 ± 0.66	0.686
Vegetables	10.55 ± 0.68	8.02 ± 1.22	13.08 ± 1.33	**0.020**
Chips & Salty Crackers	0.22 ± 0.12	0.34 ± 0.22	0.10 ± 0.24	0.533
Nuts & Seeds	0.99 ± 0.27	2.07 ± 0.49	0	N/A
Dairy Products				
Milk	1.50 ± 0.22	1.06 ± 0.39	1.94 ± 0.43	0.203
Milk Derivatives	9.02 ± 0.52	7.70 ± 0.93	10.34 ± 1.02	0.110
Milk Sweetened	0.30 ± 0.15	0.32 ± 0.27	0.28 ± 0.29	0.935
Meat, Processed Meat, Poultry, Fish, Eggs				
Red Meat	7.54 ± 0.65	8.75 ± 1.16	6.33 ± 1.27	0.242
Processed Meat	0.51 ± 0.19	0.55 ± 0.33	0.46 ± 0.37	0.890
Poultry	3.19 ± 0.42	3.35 ± 0.75	3.03 ± 0.82	0.813
Fish	0.45 ± 0.13	0.49 ± 0.22	0.40 ± 0.25	0.823
Eggs	0.82 ± 0.20	0.75 ± 0.36	0.89 ± 0.39	0.825
Fruits, Total				
Whole Fruits	7.38 ± 0.42	6.49 ± 0.74	8.26 ± 0.81	0.180
Fresh Juices (100% fruit juices)	0.26 ± 0.08	0.23 ± 0.14	0.30 ± 0.15	0.800
Sweets & Added Sugars				
Sweets	4.09 ± 0.53	5.05 ± 0.95	3.12 ± 1.04	0.250
Added Sugars, Jams, Honey, Molasses	1.49 ± 0.16	1.43 ± 0.28	1.54 ± 0.31	0.828
Sugar Sweetened Beverages	1.55 ± 0.21	1.48 ± 0.37	1.62 ± 0.41	0.835
Hot Beverages (Coffee, Tea)	0.23 ± 0.02	0.23 ± 0.04	0.24 ± 0.04	0.885
Alcoholic Beverages	1.10 ± 0.19	1.62 ± .34	0.58 ± 0.37	0.087
Added Fats & Oils	5.70 ± 0.44	6.65 ± 0.79	4.75 ± 0.87	0.18
Fast Food	0.41 ± 0.22	0.44 ± 0.39	0.38 ± 0.42	0.930
Miscellaneous	0.97 ± 0.18	1.03 ± 0.33	0.91 ± 0.36	0.838

**Abbreviations:***EI* energy intake; *SE* standard error

Values in this table represent mean ± SE

Means were adjusted for variables found to be significantly associated with gender, as per Table 1

^*^p values were obtained from comparison of adjusted means using ANCOVA

**Bolded** numbers are significant at *p* < 0.05

Micronutrients’ intakes in the study population, in terms of means ± SE and proportions of participants below 2/3rd of the RDA, are presented in Table 4. Among children and adolescents, the proportions of participants below 2/3rd of the RDA were 77.3% for calcium, 36.4% for iron, 26.6% for zinc, 55.3% for vitamin A, 26.2% for vitamin C, and 35.7% for vitamin B12. In this age group, except for vitamins A and C, the proportions of participants falling below 2/3rd of the RDA were significantly higher among females than males for the micronutrients considered in this study. Similar results were obtained when the data for adolescents (aged 12–19.9 years) were analyzed separately (Appendix B-Table 3). For adults (20–59.9 years), females had significantly lower mean intakes of all micronutrients, and consequently the proportions of females consuming less than 2/3rd of the RDA were higher than males, except for vitamin A and vitamin C where no statistical difference was noted. Among older adults (age ≥ 60 years), the proportions consuming below 2/3rd of the RDA for calcium, iron, zinc, vitamin A, vitamin C, and vitamin B12 were 79.2, 29, 42, 67.5, 45.1, and 50% respectively. The proportions of females consuming less than 2/3rd of the RDA was significantly higher than those of males for calcium and iron (Table 4).

**Table 4:** Micronutrient intake (expressed as means ± SE and percentage consuming <2/3rd RDA) by gender (n = 3394)

	Total (*n* = 3394)	Males (*n* = 1578)	Females (*n* = 1816)	p-value^*^
Children and adolescents (6–19.9 years)				
Calcium (mg)				
Mean ± SE	593.67 ± 12.4	657.35 ± 17.65	529.98 ± 17.57	**< 0.001**
Below 2/3rd RDA, n (%)	669 (77.3)	311 (71.2)	358 (83.6)	**< 0.001**
Iron (mg)				
Mean ± SE	9.93 ± 0.21	11.05 ± 0.3	8.81 ± 0.3	**< 0.001**
Below 2/3rd RDA, n (%)	315 (36.4)	108 (24.7)	207 (48.4)	**< 0.001**
Zinc (mg)				
Mean ± SE	7.84 ± 0.15	8.88 ± 0.22	6.8 ± 0.21	**< 0.001**
Below 2/3rd RDA, n (%)	230 (26.6)	87 (20)	143 (33.4)	**< 0.001**
Vitamin A (RAE, μg)				
Mean ± SE	788.4 ± 68.91	931.87 ± 98.1	644.93 ± 97.63	**0.039**
Below 2/3rd RDA, n (%)	478 (55.3)	238 (54.6)	240 (56.1)	0.660
Vitamin C (mg)				
Mean ± SE	82.86 ± 2.43	87.14 ± 3.46	78.59 ± 3.45	0.082
Below 2/3rd RDA, n (%)	226 (26.2)	108 (24.8)	118 (27.7)	0.329
Vitamin B12 (μg)				
Mean ± SE	3.63 ± 0.35	4.58 ± 0.5	2.68 ± 0.5	**0.008**
Below 2/3rd RDA, n (%)	309 (35.7)	130 (29.7)	179 (41.8)	**< 0.001**
Adults (20–59.9 years)				
Calcium (mg)				
Mean ± SE	603.48 ± 7.93	669.8 ± 13.02	537.16 ± 11.32	**< 0.001**
Below 2/3rd RDA, n (%)	1392 (66.1)	525 (56.7)	867 (73.5)	**< 0.001**
Iron (mg)				
Mean ± SE	10.68 ± 0.14	12.13 ± 0.23	9.23 ± 0.2	**< 0.001**
Below 2/3rd RDA, n (%)	948 (45.0)	125 (13.5)	823 (69.8)	**< 0.001**
Zinc (mg)				
Mean ± SE	8.8 ± 0.12	10.18 ± 0.2	7.41 ± 0.18	**< 0.001**
Below 2/3rd RDA, n (%)	698 (33.2)	245 (26.5)	453 (38.4)	**< 0.001**
Vitamin A (RAE, μg)				
Mean ± SE	842.36 ± 47.0	876.34 ± 77.18	808.37 ± 67.1	0.536
Below 2/3rd RDA, n (%)	1339 (63.6)	608 (65.7)	731 (62.0)	0.084
Vitamin C (mg)				
Mean ± SE	83.44 ± 1.56	91.84 ± 2.56	75.04 ± 2.22	**< 0.001**
Below 2/3rd RDA, n (%)	850 (41.4)	383 (41.4)	467 (41.4)	0.972
Vitamin B12 (μg)				
Mean ± SE	5.0 ± 0.37	5.85 ± 0.6	4.06 ± 0.53	**0.038**
Below 2/3rd RDA, n (%)	908 (43.1)	327 (35.3)	581 (49.3)	**< 0.001**
Older adults (≥60 years)				
Calcium (mg)				
Mean ± SE	541.31 ± 14.52	567.81 ± 26.69	514.82 ± 27.15	0.243
Below 2/3rd RDA, n (%)	336 (79.2)	162 (75.3)	174 (83.3)	**0.045**
Iron (mg)				
Mean ± SE	9.22 ± 0.33	10.08 ± 0.6	8.36 ± 0.61	0.090
Below 2/3rd RDA, n (%)	123 (29.0)	46 (21.4)	77 (36.8)	**< 0.001**
Zinc (mg)				
Mean ± SE	7.41 ± 0.2	8.39 ± 0.37	6.42 ± 0.38	**0.002**
Below 2/3rd RDA, n (%)	178 (42.0)	85 (39.5)	93 (44.5)	0.301
Vitamin A (RAE, μg)				
Mean ± SE	641.17 ± 63.27	678.58 ± 116.36	603.77 ± 116.36	0.705
Below 2/3rd RDA, n (%)	286 (67.5)	156 (72.6)	130 (62.2)	**0.023**
Vitamin C (mg)				
Mean ± SE	77.75 ± 4.14	75.85 ± 7.61	79.65 ± 7.73	0.769
Below 2/3rd RDA, n (%)	186 (45.1)	112 (52.1)	74 (37.6)	**0.003**
Vitamin B12 (μg)				
Mean ± SE	3.57 ± 0.54	4.35 ± 1.0	2.79 ± 1.01	0.356
Below 2/3rd RDA, n (%)	212 (50.0)	102 (47.4)	110 (52.6)	0.286

**Abbreviations:***μg* micrograms; *mg* milligrams; *RAE* Retinol Activity Equivalents; *RDA* Recommended Daily Allowance; *SE* standard error

Values in this table represent mean ± SE for continuous variables and n (%) for categorical variables

Means were adjusted for variables found to be significantly associated with gender, as per Table 1

^*^p-values were obtained from comparison of adjusted means using ANCOVA for continuous variables and from chi square for categorical variables

**Bolded** numbers are significant at *p* < 0.05

## Discussion

Our study adopted a life course approach to investigate sex-based differentials in dietary intakes and food consumption patterns in Lebanon, a country where inequalities in the prevalence of obesity and micronutrient deficiencies were reported between sexes. Two consistent observations across the lifespan were the higher intake of energy in males compared to females and the higher intake of dietary fiber in females compared to males. Sex-based differences in macronutrients’ intakes (% EI) and food consumption patterns were also observed. Females had a higher intake of total fat and a higher consumption of fruits, vegetables, milk, and sweets (% EI). On the other hand, males were found to have higher consumptions of red and processed meat, bread, fast food, soft drinks, and alcohol. Another consistent observation was the lower micronutrient intakes in females compared to males, across the lifespan.

The observed higher intakes of energy in males compared to females from all age groups are consistent with the well-established sex-based disparities in energy intakes, which reflect differences in physiological and metabolic factors [4, 33]. Dietary fiber intakes ranged between 6.9 and 11.6 g/1000 kcal, highlighting a gap between the population’s current intake levels and nutritional recommendations (14 g/1000 kcal), in all age groups and in both sexes. However, a consistent observation was the significantly higher dietary fiber intake in females compared to males across the lifecycle. In agreement with our findings, a review by Kiefer et al. [34] concluded that children, adolescents, and adult females tend to consume more dietary fiber compared to their male counterparts. In fact, women and girls have been frequently described as being more health conscious and more inclined to comply with dietary recommendations than their male counterparts [9, 35–38]. However, in contrast to our findings, a recent study conducted in Tunisia did not report any significant differences in dietary fiber intake between males and females [5], while a study conducted in the United Kingdom (UK) showed that, compared to men, women were significantly more likely to have an inadequate fiber intake [4].

In our study, total fat intake ranged between 35.2 and 37.7% EI, while saturated fat ranged between 10.4 and 11.2% EI, thus exceeding, in all age groups and in both sexes, the World Health Organization (WHO) upper limit of 35% EI for total fat and 8–10% EI for saturated fat [39]. However, significant sex-based differences in the macronutrients’ contributions to energy intake were observed. More specifically, adolescent girls and adult women (20–60 years) were found to have significantly higher intakes of total fat compared to males, but these differences were not observed in younger children nor in older adults. Noteworthy is the higher intake of saturated fat that was also observed among adult women compared to men. Similar to our findings, the 2012 National Diet and Nutrition Survey in the UK reported that women consume more fat and saturated fat than their male counterparts [40]. A prospective study conducted on a large sample of adults in the UK has also shown that, compared to men, women were more likely to have intakes that exceeded recommendations for total fat and saturated fat [4]. Our findings contrast with data stemming from 22 European countries [9], as well as from Tunisia [5], which did not report any significant sex-based differences in total fat intake. Our findings are also in contrast with studies reporting women as often choosing lower fat foods in an effort to make heathier food choices [9, 34].

In line with the observed differences in macronutrients’ intakes, sex-based disparities in food consumption patterns were observed. Our results underline a higher intake of fruits, vegetables, and milk in females compared to men, while showing that men had a higher consumption of soft drinks, red meat, fast food, bread, and alcohol. These findings are in agreement with previous studies conducted in Lebanon showing that males were more likely to adhere to the Western dietary pattern, while females had higher adherence to the Traditional Lebanese dietary pattern, which has been described as a variant of the Mediterranean diet with fruits, vegetables, and dairy consumption being among its characteristic hallmarks [22, 23, 41, 42]. Our findings are also in agreement with those reported from studies conducted in other parts of the world [6, 9, 43]. For instance, in Tunisia, another country undergoing the nutrition transition, women were found to consume more fruit and less soft drinks and red meat compared to men. Earlier studies have described significant gender differences in opinions and behaviors related to dietary and food choices [9]. These studies reported that men choose fewer high-fiber foods, eat less fruits and vegetables, while consuming more soft drinks and high starch foods such as bread, while women tend to consume a higher number of portions of fruits and vegetables compared to men [6, 9, 43–50]. Fruits and vegetables are rich sources of dietary fiber, antioxidants, and phytochemicals and hence dietary guidelines and recommendations have consistently encouraged a higher consumption of these food groups [51, 52]. The recent Global Burden of Disease Study (GBD) [53] showed that the optimal intake of fruit in relation to all-cause mortality falls within the range of 200–300 g/day and the optimal intake of vegetables between 290 and 430 g/day. Thus in our study, despite females having higher intakes of fruits (and of vegetables in the elderly age group) than males, their intake levels ranging between 102 and 136 g/day for fruits and between 166 and 188 g/day for vegetables (data not shown) remain low in comparison with optimal intake levels [53]. In addition, despite the significantly lower intake of red meat in females compared to males (except among the elderly age group), the consumption of red meat among Lebanese girls and women was not low compared to dietary recommendations, unlike what is usually reported from developing countries [8]. In fact, red meat intakes in females, which ranged from 27 to 32 g/day (vs 52–60 g/day for males) was within the upper limit specified by the GBD study (18–27 g/day) (data not shown) [53]. The fact that, in our study, women had a higher intake of sweets than men is also in line with findings reported by previous studies [5, 54], which have argued that men tend to prefer “hot hearty” food such as steak, while women tend to prefer sweet snacks, such as chocolate and ice cream [54]. Sweets have in fact been suggested to be culturally associated with femininity [55], while meat consumption is far more commonly associated with masculinity [56]. The observed higher sweets intake may explain the fact that a significantly higher proportion of females exceeded the upper limit of total sugar intake (25% EI) compared to men (data not shown), although the average total sugar intake did not differ between both sexes. Sweets can also be an important source of “hidden fat”, which may also explain the observed higher intakes of fat and saturated fat in women compared to men.

In agreement with evidence stemming from developing countries [8], the present study showed that women and girls in Lebanon have lower micronutrient intakes compared to their male counterparts and that higher proportions of females may be at risk of micronutrient inadequacies, including calcium, iron, zinc, and vitamin B12. In contrast to our findings, data stemming from Western societies have documented strong similarities in micronutrient intakes between both sexes [9]. The micronutrient inadequacies characterized in our study are in line with the priority micronutrients identified by the WHO for children and women of childbearing age in the EMR [57] and with those documented in previous studies in Lebanon. For instance, a cross-sectional survey by Al Khatib et al. of 470 Lebanese women (aged 15–45 years), reported that 16% were anemic (Hb < 12 g/dL), 27.2% were iron deficient (ferritin < 15 μg/L), and 7.7% were diagnosed with iron deficiency anemia [58]. In our study, the observed high proportions of women and girls not meeting 2/3rd of the RDA for several micronutrients is of concern given the impact that micronutrient inadequacies may have on physiological performance, risk for chronic disease [59–61], as well as the cycle of micronutrient inadequacies across the life span [59]. Importantly, women and girls with these nutritional inadequacies and deficits are likely to have lower educational attainment and work capability, thus contributing to further inequalities in social roles between genders [59].

Existent gender inequities and differences in social roles may also contribute to disparities in food and dietary intakes and may, at least partly, explain the observed sex-based dietary differences in our study. Despite its modernized flavor, Lebanon remains a patriarchal culture with a strong set of traditional social values, where boys tend to be given entitlement over their sisters from early childhood [62]. In a study conducted in Lebanon about perceptions of the ideal woman, the most frequently mentioned attributes, were ‘being a good housewife and mother’, ‘sacrificing’, ‘devoted to her family’, whereas attributes related to a woman’s personality or education were rarely cited [63]. These culturally dominant attributes and social roles may in fact influence the eating behavior of women and girls in Lebanon. For instance, women who do not work outside the home or who are mostly in charge of preparing meals in the household may receive more food stimuli than men, including stimuli for unhealthy foods such as sweets [5, 64]. Women may also “sacrifice” by eating less from certain foods groups, in favor of their children. A recent study by Jomaa et al. (2017) showed that, in an effort to ensure that their children are well-fed, Lebanese mothers may consume less animal-based foods such as meats and may thus have an inadequate intake of several micronutrients including iron, zinc and vitamin B12 [65, 66]. Other factors such as lower income, insufficient professional insertion, and limited decision-making within the household may also contribute to differences in dietary choices between women and men, and between boys and girls in Lebanon [67].

Overall, this study identified and characterized sex-based differences in dietary intakes and food consumption patterns in a nutrition transition context. Despite the favorable observation of higher intakes of dietary fiber, fruits, and vegetables in females compared to males, dietary intakes in women and girls remained inadequate in several micronutrients of public health concern while also being high in total and saturated fat. These observed sex-based differences place Lebanon somewhere between developed and developing countries in terms of dietary disparities between sexes, sharing the micronutrients inequalities with developing countries, while also sharing the higher fruit, vegetables, and fiber intakes with Western societies. Of interest in our study is the fact that sex-based differences in macronutrient intakes and food consumption patterns were the most noticeable in the adolescent and adult years, a timeframe that includes women’s reproductive years. These findings highlight the need for culture-specific interventions aimed at improving nutrition for women and adolescent girls as this would lay the foundation not only for their future education, productivity, and economic empowerment, but also for the health of future generations [1]. The study findings have also highlighted higher intakes of Westernized “modern” foods in males compared to females, including higher intakes of soft drinks, red and processed meat, and alcohol. Adherence to a Westernized diet has been associated with increased risk for obesity and several non-communicable diseases, thus highlighting the need for context-specific interventions aimed at promoting healthier dietary patterns in Lebanese boys and men [22, 23, 41, 42].

The present study has several strengths. The study is based on an individual food consumption survey that was conducted on a nationally representative sample of children, adolescents, and adults. The survey has covered a wide age range, permitting to examine sex-based disparities in dietary intakes across the lifespan. To reduce judgmental communication and minimize social desirability bias, the 24-HRs assessment was administered by trained nutritionists who received extensive training prior to data collection. The results of the study ought however to be considered in light of the following limitations. First, the investigation of food consumption and nutrient intakes was based on the collection of one 24-HR, which may not be representative of dietary intakes at the individual level. However, despite its well-known limitations, such as reliance on memory and day-to-day variation, the 24-HR may provide accurate estimates of energy intake and nutrient intakes at the population level [68]. In the present study, dietary information was collected by the multiple pass 24-HR approach, which was shown to improve the accuracy of dietary intake estimates in children, adolescents, and adults [69–71]. Second, it is important to mention that, in Lebanon, there exists no food composition database. Therefore, the USDA database of the Nutritionist Pro was used for dietary analysis including that of traditional dishes and mixed recipes. Although this limitation could have affected the absolute estimates of nutrient intakes, it would have had limited effects on sex-specific differences. Lastly, some of the observed differences, though statistically significant, were rather small in magnitude and hence their clinical implications may be limited.

## Conclusion

Based on an individual national nutrition survey conducted in Lebanon, this study identified sex-specific priorities that ought to be tackled by nutrition interventions aimed at promoting healthier dietary patterns and improving nutrition in both sexes. The fact that, in our study, disparities in food consumption patterns and nutrient intakes were mostly noticeable in the adolescent and adult years, and hence women’s reproductive years, call for concerted efforts to improve the diets of women and girls, putting more emphasis on the quality of food consumed and not only its quantity. Acknowledging that gender inequality crosscuts with inadequate health care, insufficient education, and limited income, sex-based differences in diet and food consumption ought to be placed at the heart of the United Nations’ SDGs on gender equality and women’s empowerment, nudging policy makers to recognize that ending hunger cannot be achieved unless all women can consume enough food with adequate nutrients [72].

## Data Availability

The datasets used and/or analyzed during the current study are available from the corresponding author on reasonable request.

## Appendix

**Table 5:** Food items included in each of the food groups considered in this study

Food Groups	Composition
**Bread**	All kinds of bread
**Cereals**	Cereals, rice, rice-based dishes, pasta, bulgur, pizza
**Legumes**	All kinds of legumes, legume-based dishes
**Starchy Vegetables**	Potatoes (including potato-based dishes), pumpkin, sweet corn
**Vegetables**	Raw vegetables (including all kinds of salads & all kinds of vegetables), cooked vegetables, vegetable-based traditional dishes, canned vegetables (including heart of palm, carrots, tomato juice, artichoke)
**Chips & Salty Crackers**	Chips, pretzels, popcorn
**Nuts & Seeds**	All kinds of nuts and seeds (including peanuts, pine, pistachios, walnuts, flaxseed, pumpkin kernels, green almonds, coconut)
**Dairy Products**	
Milk	All kinds of milk (including infant formula)
Milk derivatives	All kinds of cheese, yoghurt, laban,yoghurt-based dishes
Milk sweetened	Milk-based puddings, frozen yoghurt, fruit yoghurt
**Meat, Processed Meat, Poultry, Fish, Eggs**	
Red meat	Meat and meat organs
Processed meat	Processed meat
Poultry	Poultry and poultry organs
Fish	All kinds of seafood
Eggs	Eggs
**Fruits, Total**	
Whole fruits	Fruits and dried fruits
Fresh juices (100% fruit juices)	All kinds of fresh fruits
**Sweets & Added Sugars**	
Sweets	Pastries, candies, biscuits, cakes, traditional sweets, ice-cream
Added sugars, jams, honey, molasses	Added sugars, jams, honey, molasses
**Sugar Sweetened Beverages**	Sweetened juices, regular soft drinks
**Hot Beverages (Coffee, Tea)**	All kinds of coffee and tea
**Alcoholic Beverages**	Gin, rum, whiskey, arak, vodka, wine, beer
**Added Fats & Oils**	Olive oil, olives, avocado, sesame butter, all kinds of oils, mayonnaise, salad dressings, animal-based fat
**Fast Food**	All kinds of fast food
**Miscellaneous**	Pickles, soups, broth

**Table 6:** Energy and macronutrient intake by gender among adolescents (12–19.9 years) (*n* = 486)*

	Total ^⁋^ (***n*** = 486)	Males ^⁋^ (***n*** = 237)	Females ^⁋^ (***n*** = 249)	*p*-value
		**Mean ± SE**		
**Energy (kcal)**	2185.63 ± 39.28	2555.37 ± 56.86	1815.88 ± 55.45	**< 0.001**
**Carbohydrate (%EI)**	51.43 ± 0.46	51.86 ± 0.67	51.01 ± 0.65	0.366
**Protein (%EI)**	13.37 ± 0.17	13.58 ± 0.25	13.16 ± 0.25	0.234
**Fat (%EI)**	36.03 ± 0.42	35.03 ± 0.61	37.02 ± 0.6	**0.020**
**Saturated fat (%EI)**	10.69 ± 0.2	10.38 ± 0.28	10.1 ± 0.28	0.125
**Oleic Acid (%EI)**	9.93 ± 0.27	10.05 ± 0.39	9.82 ± 0.38	0.684
**Linolenic Acid (%EI)**	0.13 ± 0	0.13 ± 0.01	0.13 ± 0.01	0.582
**Linoleic Acid (%EI)**	4.78 ± 0.16	4.74 ± 0.22	4.81 ± 0.22	0.820
**Total Sugar (%EI)**	12.13 ± 0.37	11.77 ± 0.54	12.5 ± 0.52	0.340
**Dietary fibers** **(g/1000 kcal)**	7.55 ± 0.19	6.89 ± 0.27	8.21 ± 0.27	**0.001**

**Abbreviations**: *EI* energy intake; *g* grams; *kcal* calories; *SE* standard error

* Only including participants with completed dietary intake, and excluding those with Kcal outliers

^⁋^ Analysis of covariance (ANCOVA) was performed after adjusting for age and governorates

**Bolded** numbers are significant at *p* < 0.05

**Table 7:** Daily food consumption (% EI) contributed by food groups according to gender, among adolescents (12–19.9 years) (*n* = 476)*

	Total ^⁋^ (*n* = 476)	Males ^⁋^ (*n* = 235)	Females ^⁋^ (*n* = 241)	*p*-value
		Mean %EI ± SE		
**Bread**	16.0 ± 0.58	17.31 ± 0.84	14.68 ± 0.83	**0.028**
**Cereals**	15.87 ± 0.88	17.52 ± 1.27	14.23 ± 1.25	0.069
**Legumes**	2.71 ± 0.39	2.43 ± 0.57	2.98 ± 0.56	0.491
**Starchy Vegetables**	4.70 ± 0.34	4.20 ± 0.48	5.21 ± 0.48	0.145
**Vegetables**	5.64 ± 0.44	4.99 ± 0.63	6.30 ± 0.63	0.148
**Chips & Salty Crackers**	3.79 ± 0.35	3.12 ± 0.50	4.46 ± 0.49	0.059
**Nuts & Seeds**	1.80 ± 0.34	0.91 ± 0.48	2.67 ± 0.48	**0.010**
**Dairy Products**				
Milk	1.06 ± 0.14	0.82 ± 0.21	1.29 ± 0.20	0.102
Milk Derivatives	6.22 ± 0.38	6.13 ± 0.54	6.30 ± 0.54	0.825
Milk Sweetened	0.11 ± 0.05	0.12 ± 0.07	0.10 ± 0.07	0.821
**Meat, Processed Meat, Poultry, Fish, Eggs**				
Red Meat	4.92 ± 0.46	5.81 ± 0.66	4.04 ± 0.66	0.061
Processed Meat	1.45 ± 0.24	2.04 ± 0.34	0.86 ± 0.34	**0.015**
Poultry	4.27 ± 0.46	3.91 ± 0.66	4.63 ± 0.65	0.441
Fish	0.86 ± 0.25	1.06 ± 0.36	0.66 ± 0.35	0.424
Eggs	0.95 ± 0.20	1.13 ± 0.28	0.77 ± 0.28	0.370
**Fruits, Total**				
Whole Fruits	3.16 ± 0.26	2.36 ± 0.38	3.97 ± 0.37	**0.003**
Fresh Juices (100% fruit juices)	0.13 ± 0.05	0.08 ± 0.07	0.17 ± 0.07	0.371
**Sweets & Added Sugars**				
Sweets	8.98 ± 0.58	7.69 ± 0.83	10.26 ± 0.82	**0.030**
Added Sugars, Jams, Honey, Molasses	1.54 ± 0.14	1.47 ± 0.20	1.61 ± 0.20	0.617
**Sugar Sweetened Beverages**	6.58 ± 0.32	6.99 ± 0.46	6.17 ± 0.45	0.202
**Hot Beverages (Coffee, Tea)**	0.13 ± 0.02	0.15 ± 0.03	0.12 ± 0.03	0.562
**Alcoholic Beverages**	0.24 ± 0.09	0.27 ± 0.14	0.22 ± 0.13	0.814
**Added Fats & Oils**	4.63 ± 0.32	4.14 ± 0.46	5.12 ± 0.45	0.133
**Fast Food**	3.35 ± 0.46	4.54 ± 0.66	2.15 ± 0.65	**0.011**
**Miscellaneous**	0.93 ± 0.11	0.81 ± 0.16	1.05 ± 0.16	0.298

**Abbreviations**: *EI* energy intake; *SE* standard error

* Only including participants with completed dietary intake, and excluding those with Kcal outliers

^⁋^ Analysis of covariance (ANCOVA) was performed after adjusting for age and governorates

**Bolded** numbers are significant at *p* < 0.05

**Table 8:** Micronutrient intake among adolescents (12–19.9 years) (*n* = 495)*

	Total ^⁋^ (n = 495)	Males ^⁋^ (***n*** = 244)	Females ^⁋^ (***n*** = 251)	*p*-value
**Calcium (mg)**				
Mean ± SE	634.28 ± 16.98	745.03 ± 24.55	523.52 ± 24.0	**< 0.001**
Below 2/3rd RDA, n (%)	377 (76.2)	160 (65.6)	217 (86.5)	**< 0.001**
**Iron (mg)**				
Mean ± SE	10.96 ± 0.31	12.64 ± 0.44	9.28 ± 0.43	**< 0.001**
Below 2/3rd RDA, n (%)	185 (37.4)	41 (16.8)	144 (57.4)	**< 0.001**
**Zinc (mg)**				
Mean ± SE	8.65 ± 0.22	10.13 ± 0.32	7.18 ± 0.31	**< 0.001**
Below 2/3rd RDA, n (%)	141 (28.5)	45 (18.5)	96 (38.2)	**< 0.001**
**Vitamin A (RAE, μg)**				
Mean ± SE	882.63 ± 102.14	1118.0 ± 147.67	647.35 ± 144.31	**0.025**
Below 2/3rd RDA, n (%)	315 (63.8)	156 (64.2)	159 (63.3)	0.844
**Vitamin C (mg)**				
Mean ± SE	81.68 ± 3.1	85.83 ± 4.49	77.52 ± 4.38	0.190
Below 2/3rd RDA, n (%)	161 (32.7)	77 (31.7)	84 (33.7)	0.629
**Vitamin B12 (μg)**				
Mean ± SE	4.35 ± 0.56	6.14 ± 0.82	2.57 ± 0.8	**0.002**
Below 2/3rd RDA, n (%)	198 (40)	71 (29.1)	127 (50.6)	**< 0.001**

**Abbreviations**: *μg* micrograms; *mg* milligrams; *RAE* Retinol Activity Equivalents; *RDA* Recommended Daily Allowance; *SE* standard error;

* Only including participants with completed dietary intake, and excluding those with Kcal outliers

^⁋^**For the analysis pertinent to the mean intake:** Analysis of covariance (ANCOVA) was performed after adjusting for age and governorates. **For chi-square analysis**: No controlling for covariates was made

**Bolded** numbers are significant at *p* < 0.05

## Abbreviations

24-HR: 24 h recall
ANCOVA: Analysis of Covariance
EI: Energy intake
EMR: Eastern Mediterranean Region
GBD: Global Burden of Disease
RAE: Retinol Activity Equivalents
RDA: Recommended Daily Allowance
SDGs: Sustainable Development Goals
SE: Standard error
SPSS: Statistical Package for Social Sciences
UK: United Kingdom
UNDP: United Nations Development Programme
USDA: United States Department of Agriculture
WHO: World Health Organization
